# Usefulness of acceleration time ratio in diagnosis of internal carotid artery origin stenosis

**DOI:** 10.1007/s10396-018-0863-4

**Published:** 2018-01-31

**Authors:** Takahito Nishihira, Hidehiro Takekawa, Keisuke Suzuki, Ayano Suzuki, Yuka Tsukahara, Kentaro Iizuka, Haruki Igarashi, Akio Iwasaki, Madoka Okamura, Koichi Hirata

**Affiliations:** 1 Stroke Division, Department of Neurology, Dokkyo Medical University, 880 Kitakobayashi, Mibu, Tochigi 321-0293 Japan; 2 Center of Medical Ultrasonics, Dokkyo Medical University, Tochigi, Japan; 3 Department of Neurology, Dokkyo Medical University, Tochigi, Japan

**Keywords:** Acceleration time ratio, Peak systolic velocity, Internal carotid artery stenosis, Digital subtraction angiography, Acoustic shadow

## Abstract

**Purpose:**

The acceleration time (AcT) ratio of the internal carotid artery (ICA) is increased in ICA stenosis. However, there are few reports that have directly compared the AcT ratio to digital subtraction angiography (DSA) findings.

**Methods:**

We evaluated 177 vessels with DSA and carotid artery ultrasonography. The AcT ratio was calculated as AcT of the ICA (ICA–AcT)/AcT of the ipsilateral common carotid artery (CCA). We evaluated the correlation of DSA–NASCET stenosis with the origin of the ICA or the peak systolic velocity (ICApsv) in the stenotic region, ICApsv/peak systolic velocity of the CCA (CCApsv), ICA–AcT, and AcT ratio. Sensitivity and specificity for stenosis ≥ 70% were calculated based on the ICApsv, ICApsv/CCApsv, ICA–AcT, and AcT ratio.

**Results:**

Using NASCET criteria, 34 vessels had 70% or greater stenosis. DSA–NASCET showed a significant positive correlation with ICApsv, ICApsv/CCApsv, ICA–AcT, and AcT ratio (*p* < 0.0001). When the cut-off value for ICApsv was set at 176 cm/s, ICApsv/CCApsv at 2.42, ICA–AcT at 0.095 s, and the AcT ratio at 1.35, the sensitivity was 97.1, 97.1, 82.4, and 97.1%, and the specificity was 94.4, 91.0, 83.2, and 83.2%, for DSA–NASCET ≥ 70%, respectively.

**Conclusion:**

The AcT ratio is a beneficial parameter for evaluating ICA stenosis as well as ICApsv and ICApsv/CCApsv.

## Introduction

Management of atherosclerotic risk factors and medications such as statins is considered for asymptomatic extracranial internal carotid artery (ICA) stenosis, but carotid artery stenting (CAS) and carotid endarterectomy (CEA) are also considered for patients with an expected long-term prognosis [1]. For symptomatic extracranial ICA stenosis, CAS and CEA are considered in addition to best medical treatment for cases of severe stenosis of 70% or more based on the North American Symptomatic Carotid Endarterectomy Trial (NASCET) [2]. Therefore, from the viewpoint of primary and secondary prevention of ischemic stroke, noninvasive and simple diagnosis of extracranial ICA stenosis is important, and carotid artery ultrasonography is widely used for diagnosing extracranial ICA stenosis.

Peak systolic velocity (PSV) is widely used for the diagnosis of extracranial ICA stenosis by carotid artery ultrasonography. When PSV is 125 cm/s or higher, stenosis of 50% or more is suspected, but when PSV is 230 cm/s or higher, stenosis of 70% or more is indicated based on NASCET [3]. In addition, ICA to common carotid artery (CCA) PSV ratio is also useful for diagnosis of extracranial ICA stenosis according to NASCET criteria [4].

However, acoustic shadows will appear on ultrasonography when there is calcification, making observation of that region difficult. Therefore, diagnosis of stenosis becomes impossible by directly measuring PSV when there is circumferential calcification of the carotid arteries.

Conversely, it is known that the acceleration time (AcT) of the peripheral artery is extended where there is stenosis, and the usefulness of diagnosing extracranial ICA stenosis has been suggested [5–7] in addition to diagnosis of restenosis after CAS [8]. However, there are a limited number of cases in which AcT was directly compared to findings of cerebral angiography in the literature [7] and, moreover, only one study has been conducted to directly compare the AcT ratio, which is calculated by dividing the AcT of the ICA by the AcT of the ipsilateral CCA, and cerebral angiography [8]. However, in their study, only patients receiving CAS were included.

Therefore, we examined the usefulness of the AcT ratio for diagnosing extracranial ICA stenosis, especially ICA origin stenosis.

## Methods

The subjects were 97 consecutive patients (mean age: 68.4 years, SD: ± 10.8, 76 men) who were hospitalized in the Department of Neurology, Dokkyo Medical University, for atherothrombotic cerebral infarction, and received both carotid artery ultrasonography and digital subtraction angiography (DSA) between April 1, 2014 and March 31, 2017. Seventeen vessels were excluded, because the internal carotid arteries were obstructed at their origins; therefore, 177 vessels were retrospectively analyzed.

HT, YT, KI, AI, HI, AS, and MO performed carotid artery ultrasonography using SSA-770A (Toshiba, Japan). Ultrasound imaging was performed with the subject lying supine, the head turned away from the side being scanned, and the neck extended.

**Fig. 1:**
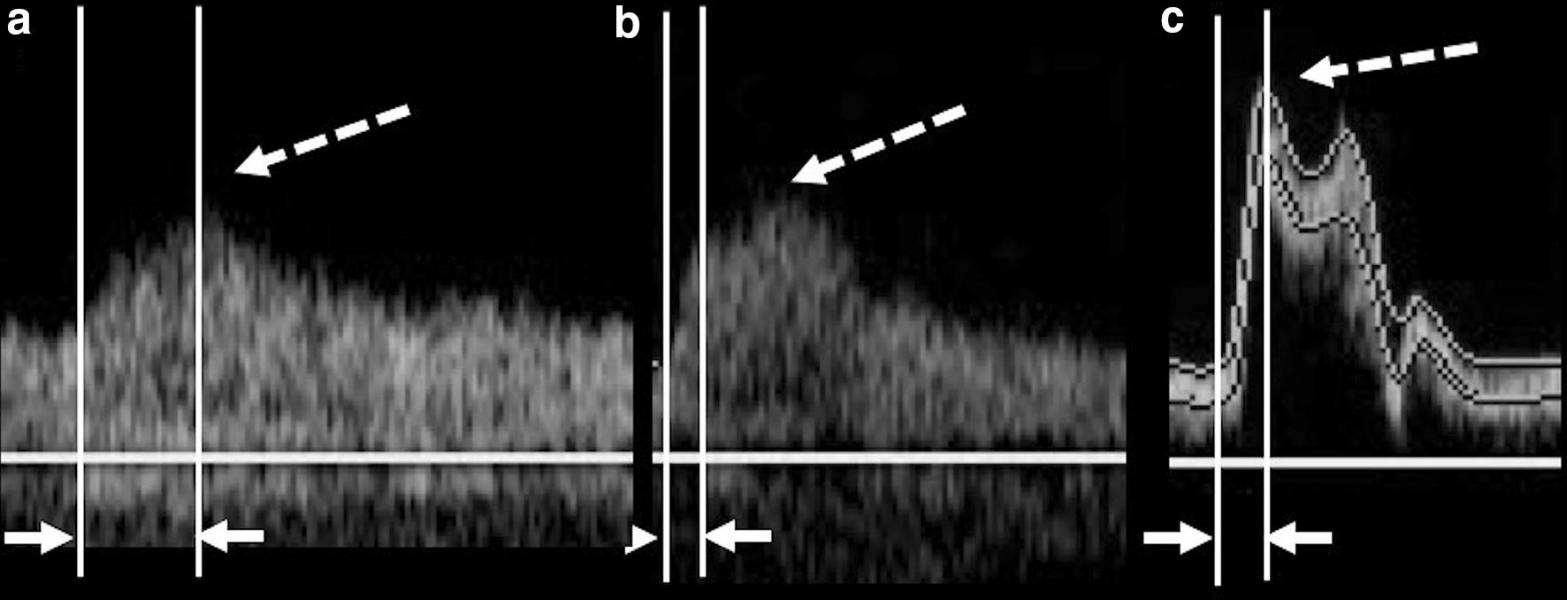
Measurement of PSV and AcT. PSV was measured at the point with maximum blood flow velocity (dotted arrows). When there was a monomodal peak pattern, AcT was defined as the time up to the maximum flow velocity (white arrows) (**a**). When there was a monomodal peak pattern with a distinct flection point, AcT was defined as the time up to the inflection point (**b**). When there were bimodal peaks, AcT was defined as the time up to the first peak (**c**). *PSV* peak systolic velocity, *AcT* acceleration time

Pulsed-wave Doppler measurement of the CCA was carried out approximately 2 cm from the carotid sinus using a linear-array probe (5–11 MHz). Pulsed-wave Doppler of the ICA was performed in the region approximately 3.5 cm (3.50 ± 0.92 cm) from the ICA bifurcation using a convex-array probe (1.9–6 MHz). The sample volume of the pulsed-wave Doppler was set at 1/2 or longer than the vessel diameter, and the Doppler insonation angle against the direction of jet flow or the blood vessel direction was 60° or smaller. In addition, power or color Doppler was used to observe the origin of the ICA, and the PSV (ICApsv) was measured after setting the sample volume in a way that sufficiently covered the stenotic lesion for cases in which stenosis was present. We measured the ICApsv at the origin of the ICA in cases in which stenosis was not present. We measured the PSV that was higher around the acoustic shadow in cases in which the point at which stenosis was most severe was unclear due to calcification. The ICApsv/CCApsv was calculated as ICApsv/(PSV of the ipsilateral CCA). The measurements of ICApsv and CCApsv were performed using pulsed-wave Doppler at the maximum peak of the waveform [4] (Fig. 1).

Measurement of AcT was carried out in accordance with the report by Takekawa et al. [6] (Fig. 1). More specifically, we showed the monomodal peak pattern and defined AcT as the time up to PSV for cases where there was no distinct inflection point. We showed the monomodal peak pattern and defined AcT as the time up to the inflection point when there was a distinct inflection point. When there were bimodal peaks, AcT was defined as the time up to the first peak. AcT, defined as time from initiation of the upstroke to the first maximum peak of the waveform, was measured by the average of five heartbeats. We calculated the AcT for the ICA (ICA–AcT) and CCA. The AcT ratio was calculated as ICA–AcT/(AcT of the ipsilateral CCA).

ICA origin stenosis diagnosed via DSA was evaluated by NT using the NASCET method (DSA–NASCET).

We evaluated the correlation of DSA–NASCET with ICApsv, ICApsv/CCApsv, ICA–AcT, and AcT ratio using the Pearson correlation coefficient, and examined whether DSA–NASCET was predictable based on each item using single regression analysis. In addition, we calculated the sensitivity and specificity for DSA–NASCET ≥ 50% and ≥ 70% based on the ICApsv, ICApsv/CCApsv, ICA–AcT, and AcT ratio using a receiver-operating characteristic (ROC) curve. Then, positive predictive value (PPV), negative predictive value (NPV), and accuracy for these parameters were calculated.

In addition, we calculated the inter-rater reliability for the ICA–AcT, AcT ratio, ICApsv, and ICApsv/CCApsv of five blood vessels using the intraclass correlation coefficients (ICC). IBM SPSS (ver. 24.0, Tokyo, Japan) was used for statistical processing and plotting, and *p* < 0.05 was considered statistically significant.

HT, KS, and HK were involved in overseeing the entire study.

## Results

### All vessels

There were 55 blood vessels (31.1%) with DSA–NASCET ≥ 50% and 34 blood vessels (19.2%) with DSA–NASCET ≥ 70%. No patient underwent CAS.The ICCs for ICApsv, ICApsv/CCApsv, ICA–AcT, and AcT ratio were 0.988, 0.996, 0.888, and 0.842, respectively.

**Fig. 2:**
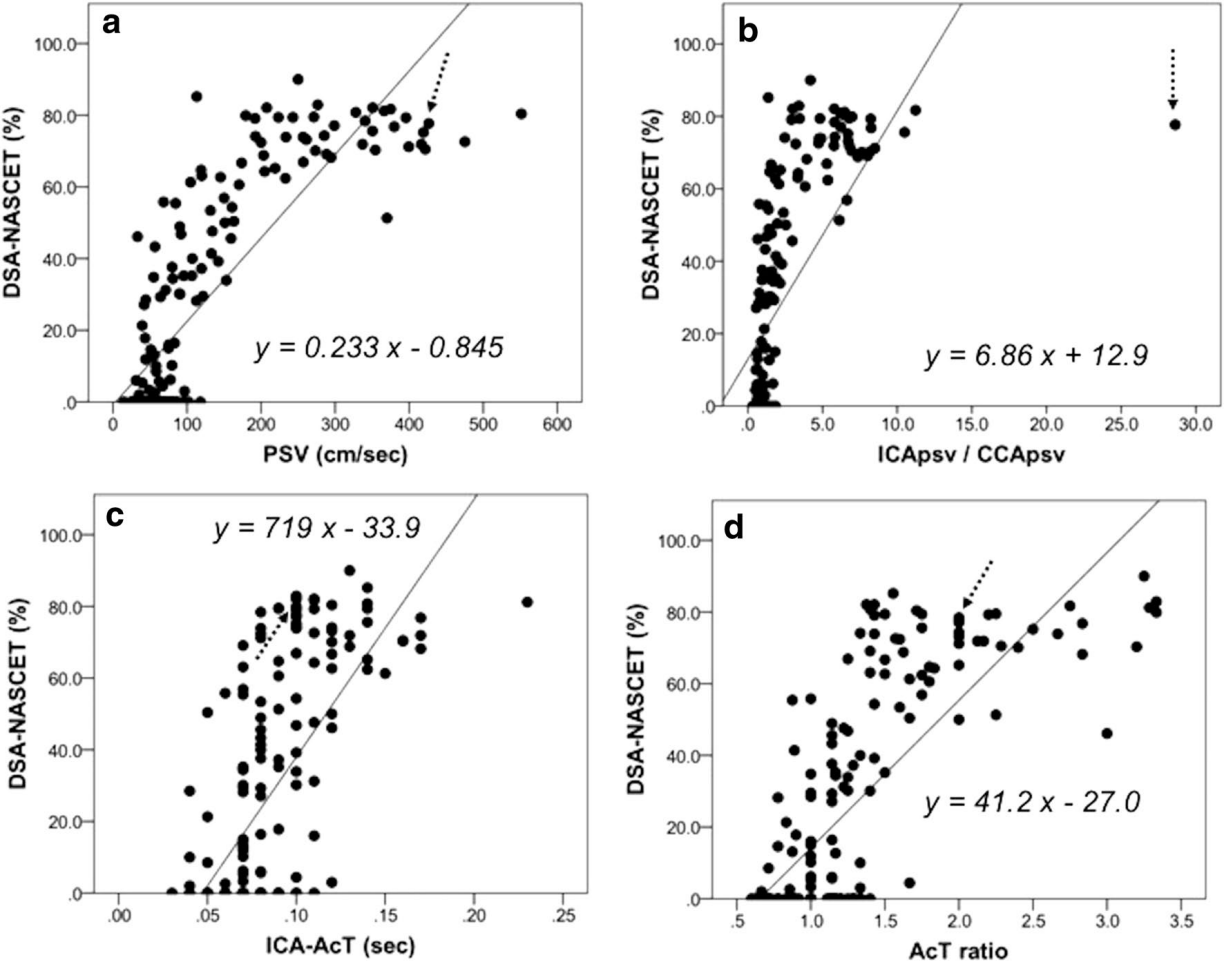
Relationship between carotid artery ultrasonography parameters and DSA–NASCET. In simple linear regression analysis, DSA–NASCET shows a positive and significant correlation with ICApsv (**a**), ICApsv/CCApsv (**b**), ICA–AcT (**c**), and AcT ratio (**d**). Dotted arrow: male, 86 years old, left carotid artery, ICA–AcT 0.1 s, AcT ratio 2.0, PSV 426.4 cm/s, ICApsv/CCApsv 28.6. *DSA* digital subtraction angiography, *NASCET* Symptomatic Carotid Endarterectomy Trial, *PSV* peak systolic velocity, *ICA*, internal carotid artery, *CCA* common carotid artery, *AcT* acceleration time. ICApsv/CCApsv = PSV of ICA/(PCV of ipsilateral CCA). AcT ratio = ICA–AcT/(AcT of ipsilateral CCA)

DSA–NASCET showed correlations with ICA–AcT (*r* = 0.647, *p* < 0.0001), AcT ratio (*r* = 0.744, *p* < 0.0001), ICApsv/CCApsv (*r* = 0.670, *p* < 0.0001), and ICApsv (*r* = 0.833, *p* < 0.0001). Furthermore, we could predict DSA–NASCET based on the ICApsv, ICApsv/CCApsv, ICA–AcT, and AcT ratio using simple linear regression analysis (Fig. 2a–d). In addition, when we excluded a vessel showing a left ICApsv/CCApsv of 28.6 (left carotid artery, Fig. 1b, dotted arrow) in an 86-year-old male patient, the correlation coefficient between ICApsv/CCApsv and DSA–NASCET increased to 0.788.

**Fig. 3:**
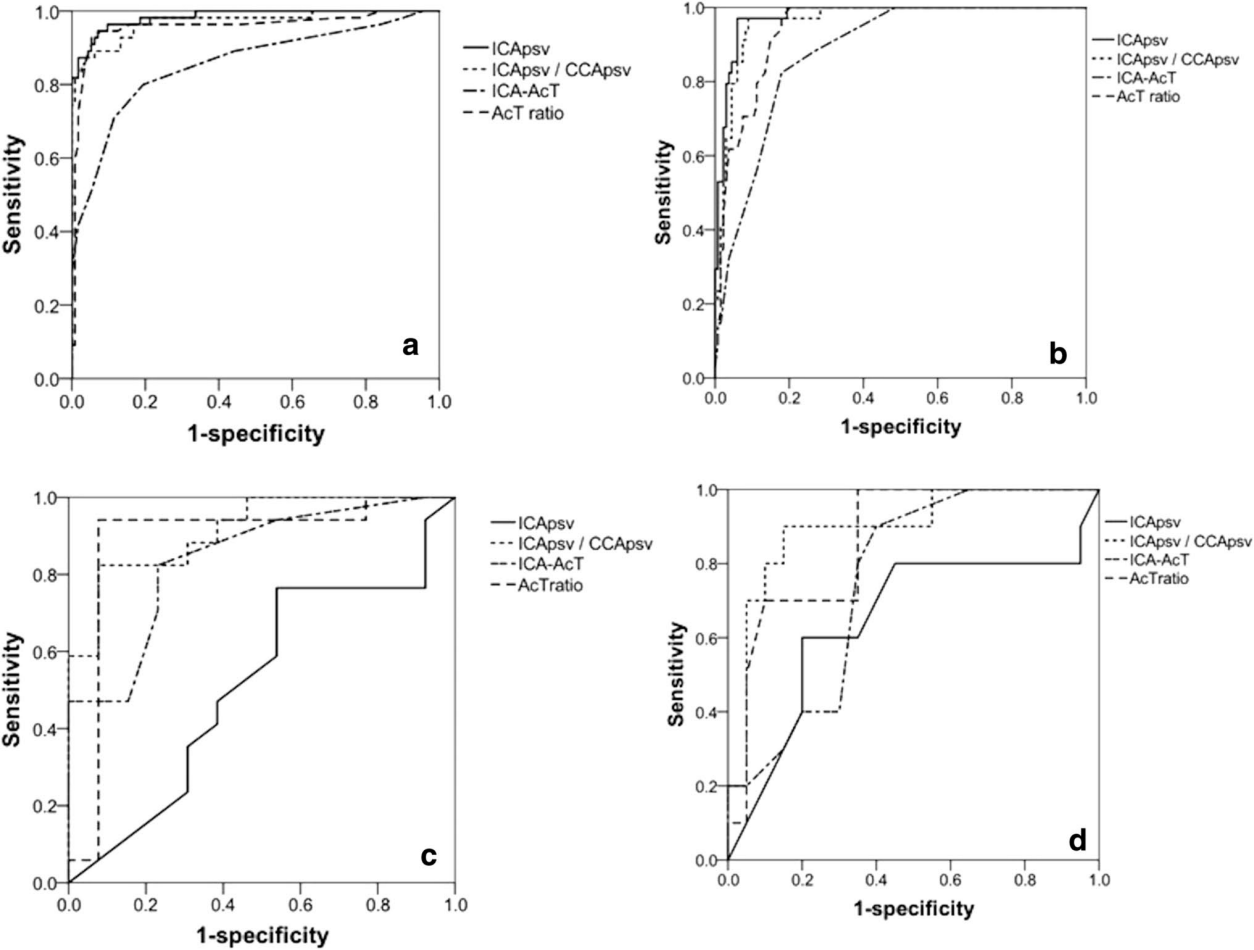
Sensitivity and specificity of DSA–NASCET stenosis according to the NASCET criteria. For predicting DSA–NASCET ≥ 50% on the ROC curve, AUC of ICApsv was 0.985, ICApsv/CCApsv was 0.970, ICA–AcT was 0.861, and AcT ratio was 0.958 (**a**). For predicting DSA–NASCET ≥ 70% on the ROC curve, AUC of ICApsv was 0.978, ICApsv/CCApsv was 0.963, ICA–AcT was 0.888, and AcT ratio was 0.945 (**b**). In contrast, in 30 vessels with acoustic shadow that interfered with direct measurement of ICApsv in the most stenotic site, for DSA–NASCET ≥ 50% on the ROC curve, the AUC of ICApsv was 0.516, ICApsv/CCApsv was 0.914, ICA–AcT was 0.839, and AcT ratio was 0.887 (**c**). For diagnosing DSA–NASCET ≥ 70% in the 30 vessels on the ROC curve, the AUC of ICApsv was 0.648, ICApsv/CCApsv was 0.895, ICA–AcT was 0.753, and AcT ratio was 0.860 (**d**). *DSA* digital subtraction angiography, *NASCET* Symptomatic Carotid Endarterectomy Trial, *PSV* peak systolic velocity, *ICA* internal carotid artery, *CCA* common carotid artery, *AcT* acceleration time, *ROC* receiver-operating characteristic, *AUC* area under curve. ICApsv/CCApsv = PSV of ICA/(PCV of ipsilateral CCA). AcT ratio = ICA–AcT/(AcT of ipsilateral CCA)

On observing the diagnostic yield for DSA–NASCET ≥ 50% on the ROC curve, the area under the curve (AUC) of ICApsv was 0.985, ICApsv/CCApsv was 0.970, ICA–AcT was 0.861, and AcT ratio was 0.958, indicating increased usefulness of the ICApsv, ICApsv/CCApsv, and AcT ratio (Fig. 3a). When the cut-off value of ICApsv was set at 112 cm/s, ICApsv/CCApsv at 1.95, ICA–AcT at 0.085 s, and AcT ratio at 1.31, the sensitivity was 94.5, 87.3, 80.0, and 94.5%, and the specificity was 93.4, 96.5, 82.0, and 91.0%, respectively. Meanwhile, the AUC for DSA–NASCET ≥ 70% was 0.978 for ICApsv, 0.963 for ICApsv/CCApsv, 0.888 for ICA–AcT, and 0.945 for the AcT ratio (Fig. 3b). In addition, when the cut-off value for ICApsv was set at 176 cm/s, ICApsv/CCApsv at 2.42, ICA–AcT at 0.095 s, and the AcT ratio at 1.35, the sensitivity was 97.1, 97.1, 82.4, and 97.1%, and the specificity was 94.4, 91.0, 83.2, and 83.2%, respectively.

## Diagnostic accuracy of the combined parameters of AcT and PSV

The diagnostic yield of DSA–NASCET ≥ 50% had slightly lower sensitivity and NPV compared with ICApsv. However, accuracy at the setting of ICApsv ≥ 112 cm/s and AcT ratio ≥ 1.31 was the highest (97.2%) (Table 1).

**Table 1:** Diagnosis rate for DSA–NASCET stenosis

	Sensitivity (%)	Specificity (%)	PPV (%)	NPV (%)	Accuracy (%)
Diagnosis for DSA–NASCET ≥ 50%					
(a) ICApsv ≥ 112 cm/s	94.5	93.4	85.2	97.4	93.2
(b) ICApsv/CCApsv ≥ 1.95	87.3	96.5	92.3	94.4	93.8
(c) ICA–AcT ≥ 0.085 s	80.0	82.0	66.7	90.1	81.4
(d) AcT ratio ≥ 1.31	94.5	91.0	82.6	97.4	92.1
Diagnostic rate of combination					
*a* + *c*	78.2	95.9	89.6	90.7	90.4
*a* + *d*	92.7	99.2	98.1	96.8	97.2
*b* + *c*	70.9	98.4	95.1	88.2	89.8
*b* + *d*	85.5	98.4	95.9	93.8	94.4
Diagnosis for DSA–NASCET ≥ 70%					
(a) ICApsv ≥ 176 cm/s	97.1	94.4	80.5	99.3	94.9
(b) ICApsv/CCApsv ≥ 2.42	97.1	91.0	71.8	99.2	92.1
(c) ICA–AcT ≥ 0.095 s	82.4	83.2	53.8	95.2	83.1
(d) AcT ratio ≥ 1.35	97.1	83.2	57.9	99.2	85.9
Diagnostic rate of combination					
*a* + *c*	79.4	95.8	81.8	95.1	92.7
*a* + *d*	94.1	95.1	82.1	98.6	94.9
*b* + *c*	79.4	95.8	81.8	95.1	92.7
*b* + *d*	94.1	93.0	76.2	98.5	93.2

ICApsv/CCApsv = PSV of ICA/PCV of ipsilateral CCA

AcT ratio = ICA–AcT/AcT of ipsilateral CCA

*DSA* digital subtraction angiography, *NASCET* Symptomatic Carotid Endarterectomy Trial, *PSV* peak systolic velocity, *ICA* internal carotid artery, *CCA* common carotid artery, *AcT* acceleration time

In contrast, the diagnostic yield of DSA–NASCET ≥ 70% was highest at the setting of 1) ICApsv ≥ 176 cm/s or 2) ICApsv ≥ 176 cm/s and AcT ratio ≥ 1.35 (94.9%). The diagnostic yield of the combination of ICApsv and AcT ratio had slightly lower sensitivity and NPV, but showed higher specificity and PPV compared to those of ICApsv only (Table 1).

## Vessels with calcification-related acoustic shadow

We identified 30 vessels with calcified plaque-related acoustic shadow that hampered direct measurement of ICApsv. Among them, 17 vessels (56.7%) had DSA–NASCET ≥ 50% and 10 vessels (33.3%) had DSA–NASCET ≥ 70%. Median calcification length was 0.9 cm (range 0.6–1.6). DSA–NASCET showed correlations with ICApsv (*r* = 0.661, *p* < 0.0001), ICApsv/CCApsv (*r* = 0.683, *p* < 0.0001), ICA–AcT (*r* = 0.639, *p* < 0.001), and AcT ratio (*r* = 0.670, *p* < 0.0001).

In contrast, on observing the diagnostic yield for DSA–NASCET ≥ 50% on the ROC curve, the AUC of ICApsv was 0.516, ICApsv/CCApsv was 0.914, ICA–AcT was 0.839, and AcT ratio was 0.887 (Fig. 3c). Meanwhile, the AUC for DSA–NASCET ≥ 70% was 0.648 for ICApsv, 0.895 for ICApsv/CCApsv, 0.753 for ICA–AcT, and 0.860 for the AcT ratio (Fig. 3d). However, the AUCs for DSA–NASCET ≥ 50% or DSA–NASCET ≥ 70% for ICApsv,ICApsv/CCApsv,ICA–AcT, and AcT ratio in the 30 vessels with acoustic shadow were smaller compared to those in all 177 vessels. Particularly, the AUC for ICApsv was smaller in these vessels.

## Discussion

The present study focused on atherothrombotic cerebral infarction, and compared DSA–NASCET to carotid artery ultrasonography, that is, ICApsv, ICApsv/CCApsv, ICA–AcT, and AcT ratio, to examine the usefulness of these indices in diagnosing the level of stenosis. Our results showed that DSA–NASCET had a significantly positive correlation with ICApsv, ICApsv/CCApsv, ICA–AcT, and AcT ratio. Based on the AUC of the ROC curve, ICApsv and ICApsv/CCApsv had the highest utility, followed by AcT ratio. Among vessels with calcified lesions, we showed the utility of ICApsv/CCApsv and AcT ratio. Furthermore, when these parameters are combined with ICApsv and AcT ratio, the diagnostic yield might be significantly improved.

Measuring the PSV in the stenotic region by pulsed-wave Doppler is known to be effective and is widely used for prediction of DSA–NASCET stenosis [3]. It has been reported that PSV > 125–150 cm/s predicts NASCET 50–69% stenosis and PSV > 200–230 cm/s predicts NASCET 70–99% stenosis [3, 9]. The utility of ICApsv/CCApsv has also been reported. ICApsv/CCApsv of 2–4 indicates NASCET 50–69% stenosis and ICApsv/CCApsv > 4 indicates NASCET 70–89% stenosis [4]. In our study, however, we determined that the cut-off values were lower than those in the previous studies. To measure PSV, it is essential to correct the Doppler insonation angle, so that it is equal to or smaller than 60 degrees [10]. However, the blood flow direction at the stenotic lesion is not always parallel to the blood vessel direction. Because of this, there are differences in the PSV and percent stenosis depending on whether the Doppler insonation angle is used as a reference or the blood vessel direction is used as a reference [11]. Therefore, we believe that cut-off values for percent stenosis diagnosis by ICApsv may vary from study to study. Similarly, cut-off values for ICApsv/CCApsv using ICApsv may differ depending on the studies. In fact, in our study, we did not have a fixed rule as to whether the Doppler insonation angle should be corrected in accordance with the blood vessel direction or the jet flow direction. In addition, we used the ICApsv that was higher around the acoustic shadow for cases where the point with the most severe stenosis was unclear due to calcification. Therefore, it cannot be ruled out that we obtained cut-off values for ICApsv or ICApsv/CCApsv similar to those in the previous studies, because we used the ICApsv at the point with the most severe stenosis.

Conversely, a few studies have reported the diagnostic yield of AcT or AcT ratio on ICA stenosis (Table 2). A part of the study by Tamura et al. [7] and the study by Kamiya et al. [8] directly compared AcT or AcT ratio and DSA–NASCET. However, the study by Kamiya et al. [8] included only the vessels in which CAS was performed. Compared to the previous reports, the cut-off value of ICA–AcT predicting stenosis was lower in our study. In general, AcT often refers to the time required to reach PSV. However,we defined AcT as the time up to the inflection point in cases with monomodal peak pattern with a distinct flection point, and we defined AcT as the time up to first peak in cases with bimodal peaks. Therefore, it is possible that shorter cut-off values for ICA–AcT were obtained in the previous studies as compared with those in the present study.

**Table 2:** Summary of previous studies evaluating stenosis and AcT/AcT ratio

Author (years)	Number of vessels	Evaluation of stenosis ratio	Parameter	Cut-off value	Sensitivity (%)	Specificity (%)	References
Takekawa et al. (2009)	127	PSV (ultrasound)	AcT	150 ms (PSV ≥ 150 cm/s)	92.3	92.1	[5]
Takekawa et al. (2009)	127	PSV (ultrasound)	AcT	160 ms (PSV ≥ 200 cm/s)	88.9	11.1	[5]
Tamura et al. (2013)	266	NASCET (ultrasound)^a^	AcT	110 ms (NASCET ≥ 50%)	58.1	88.2	[7]
Tamura et al. (2013)	266	NASCET (ultrasound)^a^	AcT	110 ms (NASCET ≥ 60%)	94.7	82.7	[7]
Tamura et al. (2013)	266	NASCET (ultrasound)^a^	AcT	110 ms (NASCET ≥ 70%)	100.0	75.9	[7]
Kamiya et al. (2015)	155^b^	PSV (ultrasound)	AcT	107.7 ms (PSV ≥ 300 cm/s)	85.7	88.4	[8]
Kamiya et al. (2015)	155^b^	NASCET (angiogram)	AcT	105.5 ms (NASCET ≥ 70%)	80.0	94.1	[8]
Present study	177	NASCET (angiogram)	AcT	85 ms (NASCET ≥ 50%)	80.0	82.0	–
Present study	177	NASCET (angiogram)	AcT	95 ms (NASCET ≥ 70%)	82.4	83.2	–
Takekawa et al. (2009)	127	PSV (ultrasound)	AcT ratio	1.75 (PSV ≥ 150 cm/s)	92.3	97.4	[5]
Takekawa et al. (2009)	127	PSV (ultrasound)	AcT ratio	2.0 (PSV ≥ 200 cm/s)	90.0	96.6	[5]
Takekawa et al. (2014)	265	ECST (ultrasound)	AcT ratio	1.5 (ECST ≥ 65%)	90.0	93.5	[6]
Kamiya et al. (2015)	155^b^	PSV (ultrasound)	AcT ratio	2.35 for PSV ≥ 300 cm/s	85.7	95.2	[8]
Kamiya et al. (2015)	155^b^	NASCET (angiogram)	AcT ratio	2.1 (NASCET ≥ 70%)	80.0	94.1	[8]
Present study	177	NASCET (angiogram)	AcT ratio	1.35 (NASCET ≥ 70%)	97.1	83.2	–
Present study	177	NASCET (angiogram)	AcT ratio	1.31 (NASCET ≥ 50%)	94.5	91.0	–

AcT ratio = ICA–AcT/AcT of ipsilateral CCA

*NASCET* Symptomatic Carotid Endarterectomy Trial, *ECST* European Carotid Surgery Trial, *PSV* peak systolic velocity, *AcT* acceleration time

^a^Angiography was performed in 11 vessels

^b^Vessels with carotid artery stenting

In the study by Tamura et al. [7], DSA was performed in 11 vessels, and mean ICA–AcT was 138.5 ± 26.3 s among the vessels with DSA–NASCET ≥ 10%. However, the AcT ratio was not shown in those vessels. DSA–NASCET and AcT ratio have not been compared in large sample studies. Takekawa et al. [6] compared AcT ratio and diameter stenosis used in the criteria of the European Carotid Surgery Trial [12] using carotid artery ultrasonography. However, the cut-off value for AcT ratio was lower in our study. An accurate evaluation of the vascular lumen is possible with DSA, but it is difficult to evaluate the vascular adventitia and the vascular endometrium without plaques. Therefore, the possibility cannot be ruled out that there was an error in the evaluation using the NASCET method, which makes use of the ratio of a normal vascular diameter and the vascular diameter of the stenotic region.

Although, in a small sample size (30 vessels), we suggest the usefulness of ICA–AcT and AcT ratio as well as ICApsv/CCApsv in vessels with calcification-related acoustic shadow that interferes with direct measurement of ICApsv in stenotic lesions. Our observation from the usefulness of ICA–AcT and AcT ratio in cases with a median length of calcified lesions of 0.9 cm suggests that ICA–AcT and AcT ratio could also be useful in cases with long calcified lesions.

There are several limitations to the present study. One is that there was no fixed rule for correction of the Doppler insonation angle. Moreover, there were cases where the ICApsv could not be measured at the point where stenosis was most severe. In addition, because evaluation using TTE was not conducted, we were unable to verify whether the influence of valvular heart disease and EF was excluded from the AcT ratio. Furthermore, the influence of ICA kinks was not evaluated. In the future, detailed investigation that takes these factors into consideration is required. Nevertheless, our study suggests a combined use of AcT ratio and other parameters will enable us to make an accurate diagnosis of stenosis rate. We believe that the usefulness of the AcT ratio as shown in the present study will be valuable in cases where the ICApsv in the stenotic region cannot be measured directly, because no study has directly compared the DSA–NASCET and the AcT ratio.

## Conclusions

We compared ICApsv, ICApsv/CCApsv, ICA–AcT, and AcT ratio to DSA–NASCET. Our results showed that the AcT ratio as well as ICApsv and ICApsv/CCApsv were highly useful. We believe that the AcT ratio is a useful evaluation method for diagnosing stenosis in cases where the ICApsv or ICApsv/CCApsv in the stenotic region cannot be measured owing to acoustic shadows caused by calcification.
